# Retraction: Application of enhanced benders decomposition algorithm in circular assembly line balancing problem with task splitting

**DOI:** 10.1371/journal.pone.0357127

**Published:** 2026-09-01

**Authors:** The PLOS One Editors

After this article [1] was published, concerns were raised regarding similarities with material previously hosted on the website of the 2023 Huawei Cloud Cup competition [2], and with a competition entry for the 2023 Huawei Cloud Cup [3] by a different research group.

Specifically:

- The Circular Assembly Line Balancing Problem with Task-Splitting problem in [1] appears similar to the circular product line and process allocation task in [2].
- Figs 2 and 4 in [1] appear similar in content to figures used in [2].
- While the article states the 60 test instances used in [1] were derived from real-world industrial projects at Huawei, [2] is not cited in [1] and the article does not clarify the association of these 60 test instances with the Huawei Cloud Cup 2023 competition.
- The Enhanced Benders Decomposition framework in [1] appears similar to the solution framework in [3], including:
  - Figs 6, 11, and 12 in [1] appear similar to figures in the competition entry defense presentation in [3].
  - Equations 32 and 34 in [1] and equations used in [3].
  - Table 4 in [1] and parts of the code in [3].

The corresponding author stated that they participated in the 2023 Huawei Cloud Cup competition. They stated they created Figs 2 and 4 in [1] and that these figures may appear similar to [2] as they describe the same circular production line setting.

Regarding the similarities between [1] and [3], the corresponding author stated that the mathematical formulations in [1] are developed from classical work on combinatorial Benders cuts, and that when Benders decomposition is applied to the same or a closely related optimization problem, the solution may naturally appear similar. They provided the underlying code for [1].

A member of the *PLOS One* Editorial Board reviewed the concerns, the author’s responses, and the provided underlying code, and stated they consider the overlap of [1] with [2] and [3] to be substantial. The Editorial Board member stated the CALBP-TS in [1] is strongly derived from, and not sufficiently distinguished from, the Huawei Cloud Cup competition task in [2]. Regarding the similarities between [1] and [3], the Editorial Board member stated that the framework in [1] does not appear sufficiently different from that in [3], that the provided underlying code for [1] has substantial structural and functional overlaps with [3], and that these code similarities are consistent with a refactoring, modularization, or adaptation of the competition-entry code, rather than an independent implementation from the same general method.

In light of the above concerns about overlap with previously published work, the *PLOS One* Editors retract this article. The retracted article [1] was removed from the *PLOS One* website at the time of retraction, due to the similarities with [2] and [3]. The article’s Copyright and Data Availability statements were also updated at that time, and the removed contents are no longer offered under the Creative Commons Attribution License.

All authors did not agree with the retraction.
